# Combined flat-field and frequency filter approach to correcting artifacts of multichannel two-photon microscopy

**DOI:** 10.1117/1.JBO.29.1.016007

**Published:** 2024-01-23

**Authors:** Thomas Knapp, Natzem Lima, Noelle Daigle, Suzann Duan, Juanita L. Merchant, Travis W. Sawyer

**Affiliations:** a University of Arizona, Department of Biomedical Engineering, Tucson, Arizona, United States; b University of Arizona, Wyant College of Optical Sciences, Tucson, Arizona, United States; c University of Arizona, College of Medicine, Tucson, Arizona, United States

**Keywords:** image artifacts, multiphoton microscopy, autofluorescence, image processing, scanning microscopy

## Abstract

**Significance:**

Multiphoton microscopy (MPM) is a useful biomedical imaging tool for its ability to probe labeled and unlabeled depth-resolved tissue biomarkers at high resolution. Automated MPM tile scanning allows for whole-slide image acquisition but can suffer from tile-stitching artifacts that prevent accurate quantitative data analysis.

**Aim:**

We have investigated postprocessing artifact correction methods using ImageJ macros and custom Python code. Quantitative and qualitative comparisons of these methods were made using whole-slide MPM autofluorescence and second-harmonic generation images of human duodenal tissue.

**Approach:**

Image quality after artifact removal is assessed by evaluating the processed image and its unprocessed counterpart using the root mean square error, structural similarity index, and image histogram measurements.

**Results:**

Consideration of both quantitative and qualitative results suggest that a combination of a custom flat-field-based correction and frequency filtering processing step provide improved artifact correction when compared with each method used independently to correct for tiling artifacts of tile-scan MPM images.

**Conclusions:**

While some image artifacts remain with these methods, further optimization of these processing steps may result in computational-efficient methods for removing these artifacts that are ubiquitous in large-scale MPM imaging. Removal of these artifacts with retention of the original image information would facilitate the use of this imaging modality in both research and clinical settings, where it is highly useful in collecting detailed morphologic and optical properties of tissue.

## 1 Introduction

**Fig. 1:**
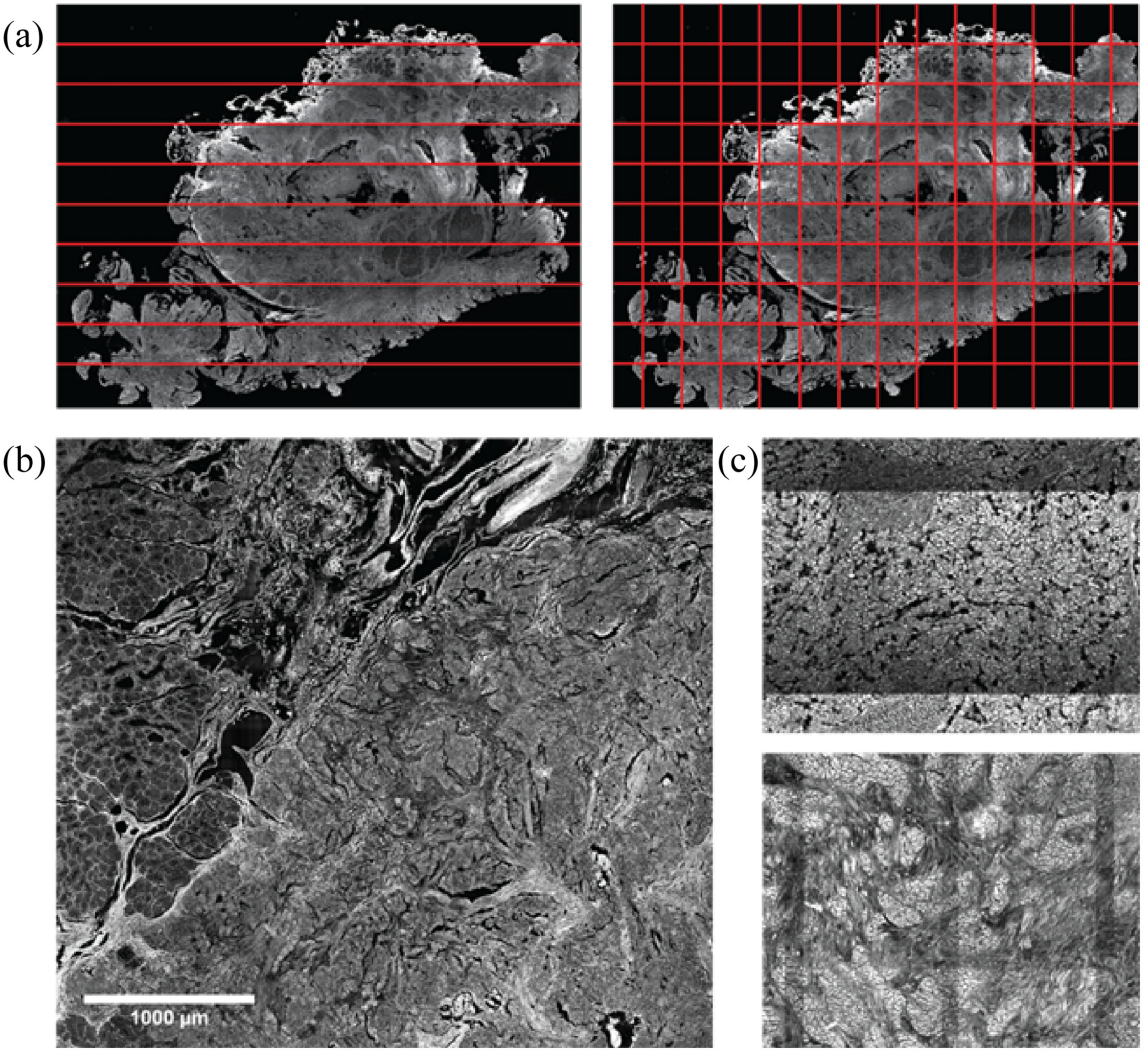
(a) Example of line scan (left) and tile scanning (right) techniques for capturing large microscope images. (b) Subregion of scanned duodenal tissue showing the border between normal tissue (left side of image) and tumor tissue on the right-hand side. Large scans allow for statistically significant quantitative analysis to be performed from the high number of samples that can be derived and directly compared within a single image. (c) Examples of different tiling artifact appearances from two separate wavelength channels. All images were collected using two-photon fluorescent microscopy with signal generated from endogenous fluorophores.

Whole-slide imaging (WSI) has gained popularity for both clinical and research applications due to the rapid acquisition of large, high-resolution, images that enable microscopic visualization of functional and structural markers in tissue samples.^1^ Traditional WSI systems utilize a motorized stage and scanning microscope to capture brightfield and/or fluorescence image data over the entire area of a sectioned specimen. Images are acquired in line arrays or tiled segments [Fig. 1(a)] with a percentage of overlap and are then stitched together to create the final composite of the full sample. Proper combination of the tile or line-scanned images requires coordination of the microscope hardware and software to eliminate artifacts that can appear between individual segments [Fig. 1(c)] due to sample nonuniformity, illumination nonuniformity, image aberrations, and vignetting, among other sources of variation. Advances in WSI have produced fast-acquisition, high-resolution systems that avoid such artifacts using proprietary technology that comes at a high cost and may not be accessible or available for many researchers.^1^^,^^2^ The inclusion of multichannel or multiphoton capabilities in commercial WSI systems exacerbates microscope costs,^3^ which limits the acquisition of large spectrums of fluorescent, autofluorescent, or second-harmonic generation (SHG) data.

An alternative to WSI systems is the use of microscopes with automatic motorized stages and software that coordinate the same tile or line array image scanning. The use of multiphoton microscopy (MPM) in such an application allows for inherently high-resolution imaging of functional or structural markers with a reduced likelihood of damaging sensitive organic samples due to the doubling of excitation wavelength, thus exposing the sample to lower incident photon energy.^4^ Collecting whole-slide MPM images can provide a robust dataset of the fluorescent and structural properties of organic samples^5^ and is particularly useful when comparing tissue types that are collected within the scan area [Fig. 1(b)]. Direct comparison of tissue and/or cell types within the same whole-slide image allows for pair-wise comparisons of optical signals while eliminating potential confounding variables such as day-to-day and image-to-image variations in system performance that can affect sensitive quantitative analysis.

These setups often lack the proprietary technology to prevent or remove the creation of artifacts that appear where tile scans overlap, which take on a grid-like appearance [Fig. 1(c)] and are a well-known phenomenon.^6^ In this paper, we will refer to this grid-like variation of brightness at the borders of adjacent tiles as a tiling artifact.

Methods to remove tiling artifacts from stitched microscopy images, using both system design and postprocessing methods, have been a popular area of study due to the utility of WSI. Early WSI systems incorporated linear array detectors to mitigate the effect of uneven sample illumination that would cause vignetting of scanned images.^6^ For MPM and confocal microscopy, z-stacking, or the collection of image tiles at increment points of focus through a sample, and the creation of a composite image from the combined z-scans can have a significant impact on data quality by reducing the effect of imperfect sectioning.^7^ This process can correct for brightness nonuniformity over large acquisition areas due to the uneven texture of the sample placing areas outside of the initial focal plane. In addition, postprocessing techniques for reducing stripe, grid, or tiling artifacts have been a wide area of research in the field of aerial imaging and microscopy. These methods include advanced image registration and stitching methods,^8^^–^^10^ flat-field (FF)-based processing,^11^ fusion-based techniques,^12^ Fourier-based filtering,^13^ shading correction,^14^ and deep learning methods.^15^

While a few tile artifact correction methods for this type of imaging are available, one major challenge is determining which, if any, of these correction methods will provide adequate correction while preserving the surrounding image data. This becomes particularly challenging in multiwavelength imaging as variations in signal intensity and contrast between tissue structures can potentially alter brightness uniformity between scan regions. Often, custom algorithms that are not open source are difficult to replicate, inhibiting reproducibility, interpretation of postprocessed data, and their use in specific applications. Furthermore, some approaches may require constraints on background noise or sample homogeneity for the processing methods to perform adequately.^12^^,^^16^ Therefore, there remains a need to assess the best generalizable approach for stitching tile scans of multichannel MPM images such that researchers interested in using this technology can incorporate and modify it for their own applications. The goal of this work was to study the performance of several basic methods for removing tile artifacts through various tile scan fusion methods, FF-based corrections, and frequency filtering, in conjunction and individually, to determine the optimal process of artifact removal with retention of the original image data. This study is a continuation of a previously published SPIE proceeding paper.^17^ Additions and modifications to the data and analysis have been made to provide a more thorough exploration of the processing methods. Specifically, additional image channels have been added, including another multiphoton autofluorescence channel and the SHG channel. The data processing was modified to remove the use of MATLAB and to instead rely solely on open-source software. The analysis was changed to include additional parameters allowing for the comparison of regions containing the tiling artifacts pre and postprocessing. Modifications were made to existing figures and additional figures were added for clarity of study methods and results. Finally, the visualization of data was changed for quick and easy comparisons between processing steps.

## 2 Methods

### 2.1 Imaging

**Fig. 2:**
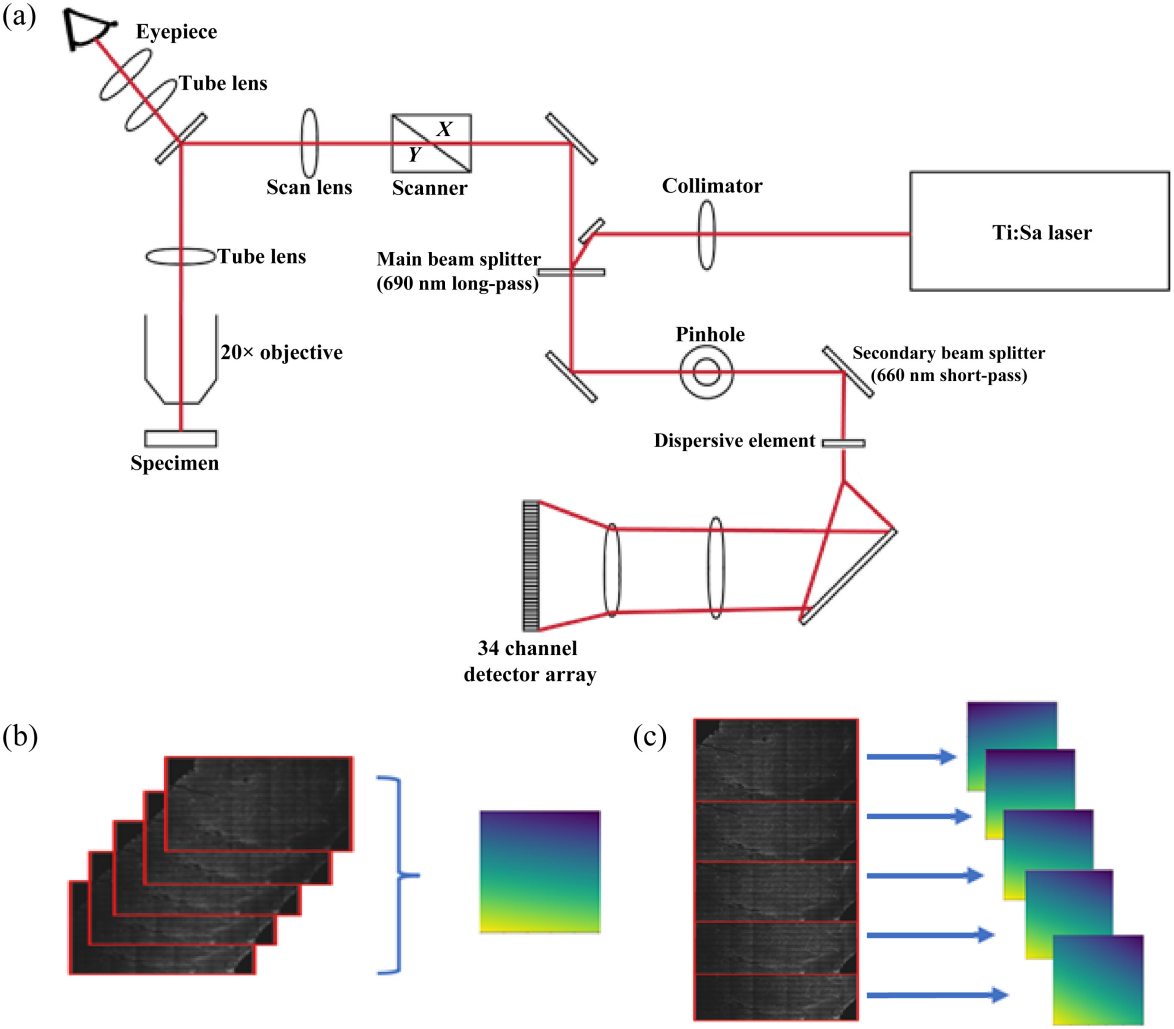
(a) Diagram of the Zeiss LSM 880 NLO upright microscope system which uses a tunable Ti:Sa light source and tunable multichannel detector array. The tunable laser and detector allows the operator to tune the system to collect signals that are predominantly from specific fluorophores, as done in this study. (b) Depiction of the cumulative FF generation approach. Each image of the z-stack has its pixel values averaged prior to the generation of the statistics-based FF. The tile images from all z-stacks are then processed using this singular FF. This process is repeated for each individual image channel. (c) Depiction of the individual FF generation approach. The images from the z-stack are used to create separate FFs using the same statistics-based approach. The tile scans of the individual z-stack images are then corrected using the corresponding FF generated from the z position of the tile.

Formalin-fixed paraffin-embedded slides of gastroenteropancreatic neuroendocrine tumors were obtained from the University of Michigan Endocrine Oncology Repository (IRB #HUM00115310) through a Materials Transfer Agreement with the University of Michigan (Dr. Tobias Else). Original tissue specimens were diagnosed as gastrinoma through positive immunohistochemistry for gastrin or documentation of patient hypergastrinemia presurgery. Informed consent was given by the patient prior to sample collection and all specimens were de-identified prior to transferring the slides. Samples were collected from the duodenum during upper endoscopy or during surgical procedure and placed in 10% neutral-buffered formalin prior to paraffin embedding and sectioning. All patient samples, and multiphoton images of the samples, included regions of tumor and adjacent normal duodenum. This was confirmed by pathology review of adjacent slides that were hematoxylin and eosin stained. In total, 11 unstained patient samples were dry-mounted and imaged with a Zeiss LSM 880 NLO upright multiphoton confocal microscope using a Plan-Apochromat 20×/0.8 M27 objective (Zeiss, White Plains, New York), tunable Titanium:Sapphire laser light source (Mai Tai HP DeepSee; Spectra-Physics, Milpitas, California) and 34-channel Quasar detector as shown in Fig. 2(a). Laser power was adjusted to 50 mW for all wavelengths with a detector gain of 800, camera dwell time of 8.19  μs, and single line averaging. Samples were imaged using five separate wavelength channels at 256×256 (1.68  μm) XY pixel resolution, with laser and detector parameters set for the acquisition of autofluorescent molecules/structures [nicotinamide adenine dinucleotide (NAD+), flavin adenin dinucleotide (FAD), and collagen] as shown in Table 1. A 690 nm long pass filter was chosen for the invisible light laser path and a 660 nm short pass filter was used for the emission light path.

**Table 1:** Excitation and emission wavelengths used in collecting autofluorescence signal of human duodenal gastrinoma tissue.

Fluorophore	Excitation wavelength (nm)	Emission wavelengths selected for detection (nm)
NADH	700±1.05	425 to 465
Porphyrin	800±1.2	590 to 625
Lipofuscin	830±1.25	550 to 600
SHG	880±1.32	430 to 450
FAD	920±1.38	475 to 600

The collection of the square mosaic image was done with the native Zeiss Zen Black software and motorized stage using a 10% tile overlap over a minimum area of 7×7 tiles for the smallest sample of tissue under study. The area of overlap between adjacent tiles can be increased for marginal reduction in artifact creation, but this adds significantly to the overall acquisition time. Table 2 shows the specific tile dimensions of the images included in the data set. A collection of 5 to 7 z-stacked images were generated with a 1- to 2-μm step size, depending on the degree of brightness nonuniformity over the scan area. Additional z-dimension scans were performed if a >50% brightness decrease remained between in-focus and out-of-focus regions through all the initial z-stack, with a minimum of three and up to a maximum of seven in this study. All fluorescent channels were saved as separate image files in 16-bit.tiff format for processing and analysis and the raw Zeiss.czi image format for comparison against postprocessed images. All images were acquired by the first author of this paper.

**Table 2:** Images used in the analysis of processing methods with their respective width×height tile and physical dimensions, each tile being 256×256  pixels at 1.66-μm pixel resolution with a 10% overlap between adjacent tiles.

Sample	Tile dimensions (width × height)	Physical dimensions (mm)
UOMA002	10×7	3.8×2.7
UOMA003	9×11	3.5×4.2
UOMA006	10×10	3.8×3.8
UOMA007	13×10	5×3.8
UOMA008	13×13	5×5
UOMA009	10×19	3.8×7.3
UOMA010	15×15	5.8×5.8
UOMA016	20×20	7.7×7.7
UOMA017	8×12	3.1×4.6
UOMA021	17×20	5.2×7.7
UOMA022	15×18	5.8×7

### 2.2 Image Processing

A custom FF-based correction was written in Python^18^^,^^19^ using a retrospective method,^20^ which did not require calibration images. This FF correction summed and normalized the pixel values of each 256×256 image tile either from the full set of z-stack images (cumulative FF correction), Fig. 2(b), or from each z-stack image independently (individual FF correction), Fig. 2(c). A line was fit to the mean of both the normalized row and column values and an FF mesh was generated using the normalized residual sum of square values for each pixel. Each image tile was then divided pixel-by-pixel by the FF matrix. FF meshes created from the individual z-stack images (instead of the entire set of images) were only used to correct for vignetting of the images from which they were generated, i.e., used to process tiles from that specific z-scan. The use of these separate methods was done to study if a corrective mesh generated from a greater range of the vignetted pixel values would have a greater performance in correcting the brightness nonuniformity and retaining image structure.

Image tiles were stitched using the ImageJ^21^ grid/collection stitching plugin^22^ with regression, max/avg displacement, and absolute displacement thresholds set to their default values of 0.3, 2.5, and 3.5, respectively. Fusion methods available in this plugin include averaging, linear blending, and maximum, minimum, or median intensity blending, which affect how pixel values are adjusted in overlapping areas of adjacent tiles. Each of these fusion types was used to determine the optimal method. An option for “subpixel accuracy” (SPA) is included in the plugin but not specifically referred to in the paper detailing the creation of the software,^22^ although it is assumed to adjust how the tile alignment is performed using linear interpolation of overlapping pixels based on the plugin source code.

Poststitched images were frequency filtered using a Python script that performed a fast Fourier transformation^23^ and selectively masked frequency peaks within the X,Y=0 or “crosshair” region of the Fourier domain data where the horizontal and vertical tiling artifacts frequencies are located.^24^ Frequency values were masked if they were greater than the mean logarithmic value within this region.

### 2.3 Quantitative Image Analysis

The 11 patient samples imaged using the four autofluorescence and the SHG channel parameters were processed using the above steps to assess its ability to correct MPM images with varying severities of tiling artifacts. Each image in the set was processed with cumulative and individual FF approaches to create two distinct FF-corrected groups. The two FF-corrected groups, and a non-FF-corrected set of the images, were then stitched using each of the fusion methods included in the ImageJ plugin with and without SPA. Each z-stack image was individually stitched and then combined using the ImageJ max projection function to correct for brightness drop-off. Raw .czi files of these images were max projected in a similar fashion without further processing to act as a baseline comparison against the processed images. Frequency filtering was used on the FF-corrected groups, the non-FF-corrected images, and the raw images to produce a dataset containing each combination of processing steps for comparison.

**Fig. 3:**
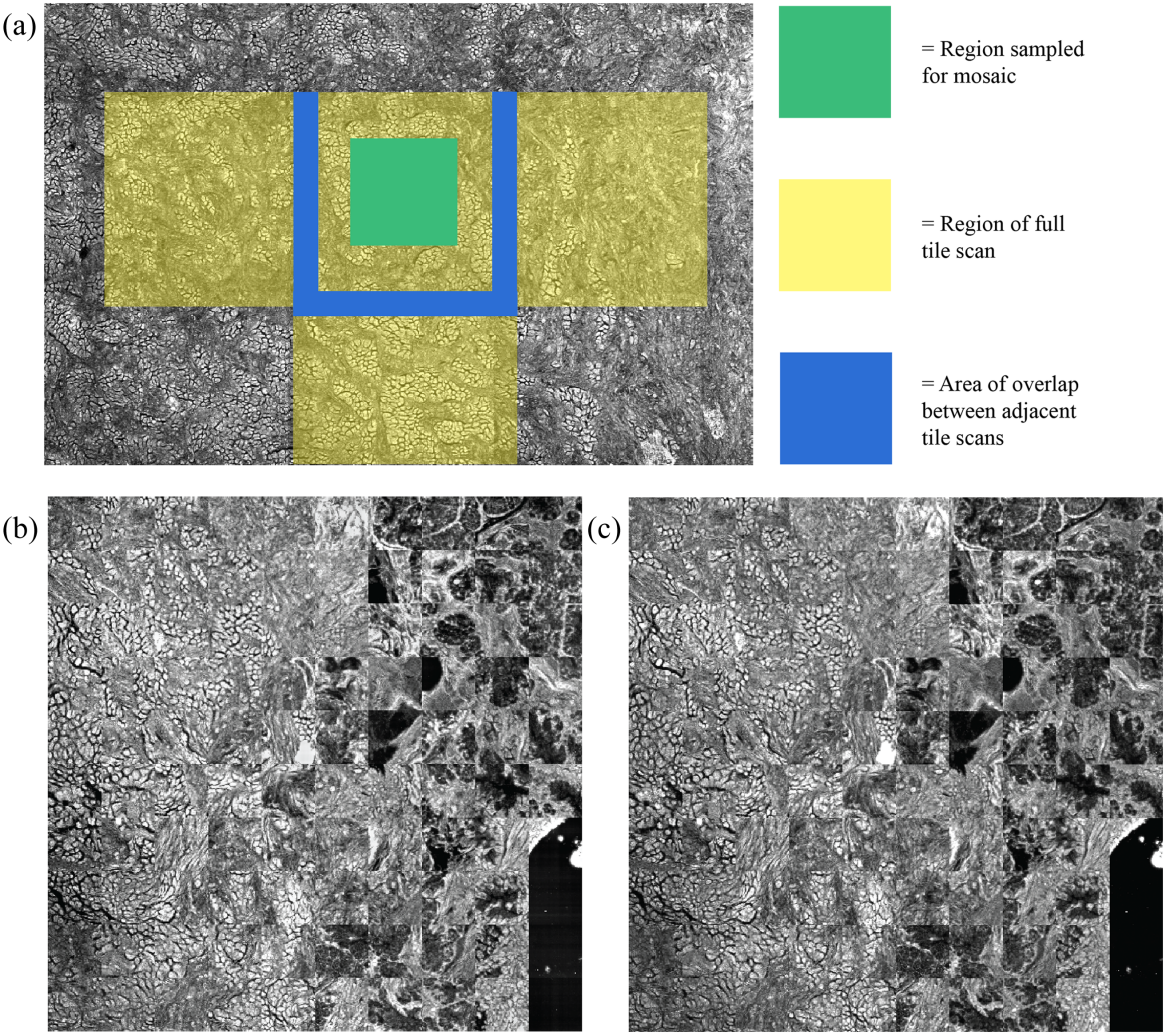
(a) Illustration of how regions were sampled from within separate tile scans for comparison pre-/postprocessing. The yellow region is the entire area of a single tile scan. The green square is the region of each tile that is taken and combined into the mosaic shown in panels (b) and (c). The blue region shows how each adjacent tile scan overlaps. (b) An image processed using the cumulative FF, with linear interpolation and linear blending of the tile overlap regions, and frequency filtering that has been transformed into a mosaic of 100×100  pixels sampled from the center of each tile scan. (c) An unprocessed image that has undergone the same transformation to measure the RMSE and SSIM between the two images without including the regions that are expected to change with removal of the tiling artifact.

The 100×100-pixel regions-of-interest (ROIs) were sampled from the center of each 256×256 tile in the processed and raw images and were compared as full ROI images for quantitative analysis as shown in Fig. 3. The selection of regions away from the tile artifacts was done as corrective measures would inflate the measured error between processed and raw image. Root mean square error (RMSE) and structural similarity index (SSIM) were used to determine how well image quality was retained away from the corrected tiling artifacts. The use of these measurements was inspired by other works on removing striping artifacts,^12^^,^^16^^,^^24^ which also allows for easy comparison against other methods.

Due to the inherent difference in image brightness between pre- and postprocessed images that would influence RMSE [Eq. (1)] and SSIM [Eq. (2)] values in this case, the kurtosis and skew of image histogram values were measured to determine how brightness uniformity was changed. Row and column (X and Y dimension) values of each image were averaged prior to generating the histograms, as these values were expected to be heavily influenced by the grid-like tiling artifacts. Values of zero were ignored during the averaging of row and column values to prevent heavy background influence in images with a large amount of dark background surrounding the sample. RMSE, SSIM, kurtosis, and skew were generated using Python^19^^,^^25^ and the values from the 11 samples were averaged into a single dataset.

RMSE is calculated using the following function:

$$\mathrm{RMSE}\;=\sqrt{\frac{\sum_{i,j=0}^{M,N}{\left(x(i,j)-y(i,j)\right)}^{2}}{M\;\times \;N}}\;,$$ (1)

where M and N are the number of rows and columns in an image, i and j are the index values of pixels x and y from the two image arrays being compared. The RMSE is a measure of difference in pixel values between the original and processed image data, thus a smaller RMSE would indicate lower error introduced by processing. The RMSE was modified into a percent error of 16-bit image data for easier interpretation of the change introduced by the processing steps using the following equation:

$$\%\mathrm{RMSE}=\frac{\mathrm{RMSE}}{65535}\times 100.$$

The SSIM was introduced by Wang et al.^26^ and compares weighted contrast, luminance, and structural information between two images as a measure of image quality retention postprocessing (e.g., compression and filtering)

$$\mathrm{SSIM}(x,y)=[l(x,y){]}^{\alpha }\times [c(x,y){]}^{\beta }\times [s(x,y){]}^{\gamma },$$ (2)

where l is luminance, c is contrast, and s is the structure comparison with weighting factors α, β, and γ that can be adjusted from 0 to 1. The SSIM was determined using with a spatial weighting of the image mean and variance using a gaussian kernel with a width of sigma=1.5 to match the implementation done by Wang et al.^26^ The value of SSIM measurements increases with greater likeness between images, with a value of 1 indicating an exact match.

Kurtosis and skew are used as a means of determining changes in the pixel value distribution and are used in this analysis to address difficulties in accurately quantifying changes that occur within artifact regions at the different stages of processing. RMSE and SSIM are unsuitable for comparing regions containing the tile artifacts since the intention of the processing steps is to generate images that are distinct from the original and creation of a true “ground-truth” at such a scale was not feasible. It is expected that processed images will have a degree of difference in comparison with the raw images, particularly from the flat-fielding which changes pixel values to correct for vignetting. Because the brightness nonuniformity causing the tiling artifact tends to be similar within each tile region for a specific channel/sample combination, we averaged the row and column values to exaggerate the repetitive brightness nonuniformity in the distribution plots. Images were masked and a threshold was used to eliminate background influence on the skew and kurtosis measurements. Skew is a measure of a distribution tail direction, e.g., a negative skew indicates that there is a greater abundance of pixels with intensities lower than the mean compared with pixels of higher intensity. Kurtosis is a measure of the size of distribution tails, with kurtosis of zero being a normal distribution and higher kurtosis indicating wider tails, or a greater amount of outlier pixel values. With the generally homogeneous autofluorescence of these tissue samples, these histogram measurements are used to indicate if the severity of vignetting causing the tiling artifact has been reduced with the processing methods by showing how the pixel values have changed from the original image and if they assume a more normal distribution.

Determination of the influence that linear interpolation (the use of subpixel accuracy) has on the tile stitching process was done through stitching the 11 sample images with and without this step for each fusion method. With 5 image channels, 5 fusion methods, and 11 patient samples, this resulted in a total sample size of 275 images stitched with and without linear interpolation for each of the three FF groups (cumulative, individual, and no FF). The fusion methods are compared pre- and postfrequency filtering of the images to determine how each step of the processing pipeline would modify image quality. Qualitative comparisons of images combined with and without linear interpolation were not included as there were no significant visual differences between the two.

## 3 Results and Discussion

**Fig. 4:**
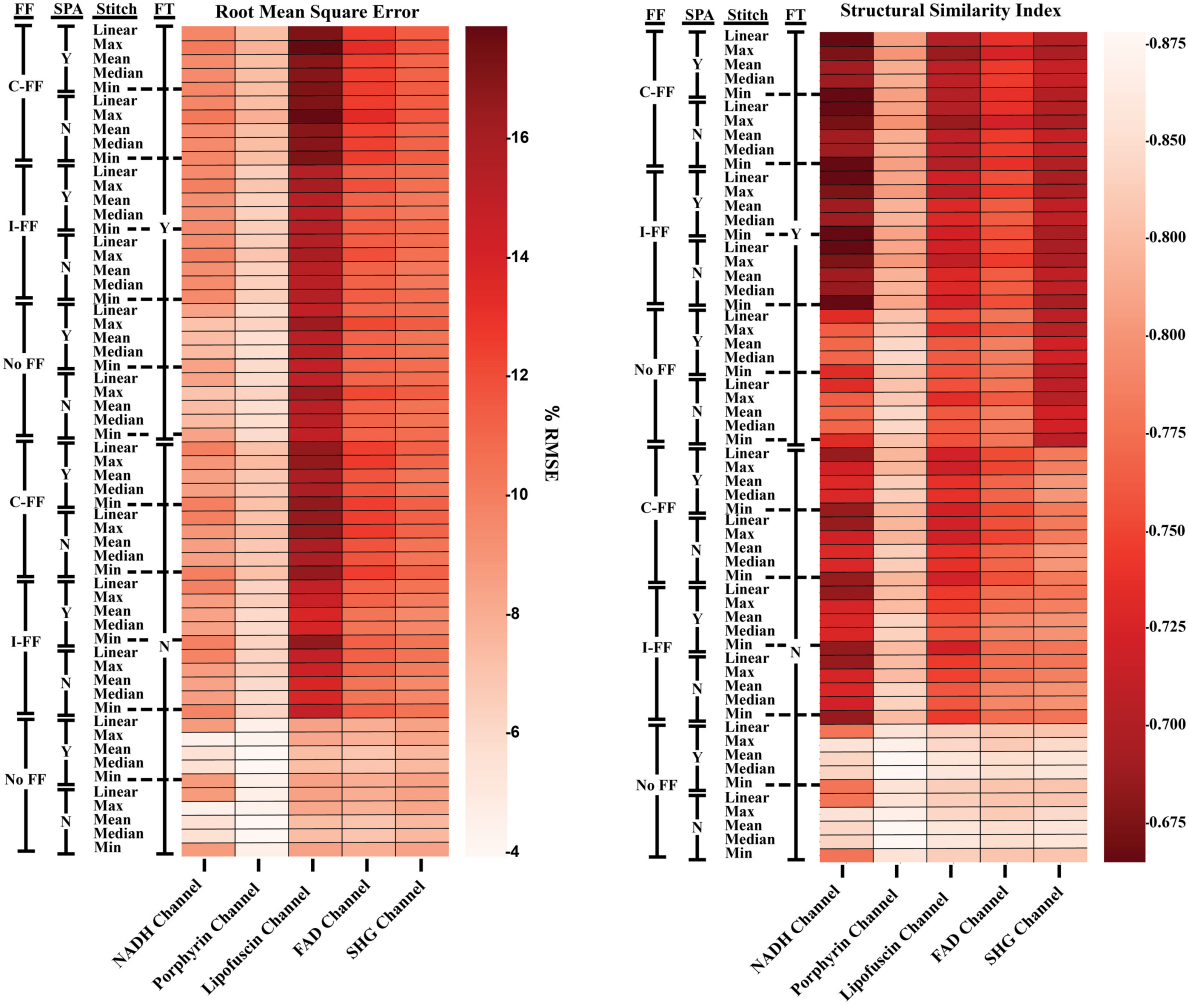
Heatmap representations of the RMSE and SSIM between raw and processed images. SPA, linear interpolation used during the process of tile registration and stitching; C-FF, tiles corrected with the cumulative FF; I-FF, tiles corrected with the FF created from the individual Z-stack images; No-FF, tiles not corrected with a FF; Y, FT filter process used; N, FT filter process not used.

Comparison of the RMSE and SSIM values of the various processing steps (Fig. 4) indicate differences in the degree of change between raw and processed images for both the different processing steps and image channels. There is a high degree of correlation between the RMSE and SSIM values (Fig. S1 in the Supplementary Material), which suggests that the percentage of error is related to differences in image texture features that would influence the SSIM. The highest SSIM values are seen in the images that have not undergone FF correction. With the effect that luminance has on SSIM, it is thought that the reduction of vignetting in the individual tiles from the FF correction had a notable impact on the similarity between raw and processed images. Normalization of the image data to floating point values between 0 and 1 results in a significantly higher value in the SSIM between pre- and postprocessed images (Fig. S2 in the Supplementary Material). This indicates that the RMSE and SSIM values are highly influenced by image brightness correction and not modifications of the original image data. The mean and median methods for combining image tiles appear to have improved RMSE and SSIM values in comparison with the linear, max, and minimum combination processes.

**Fig. 5:**
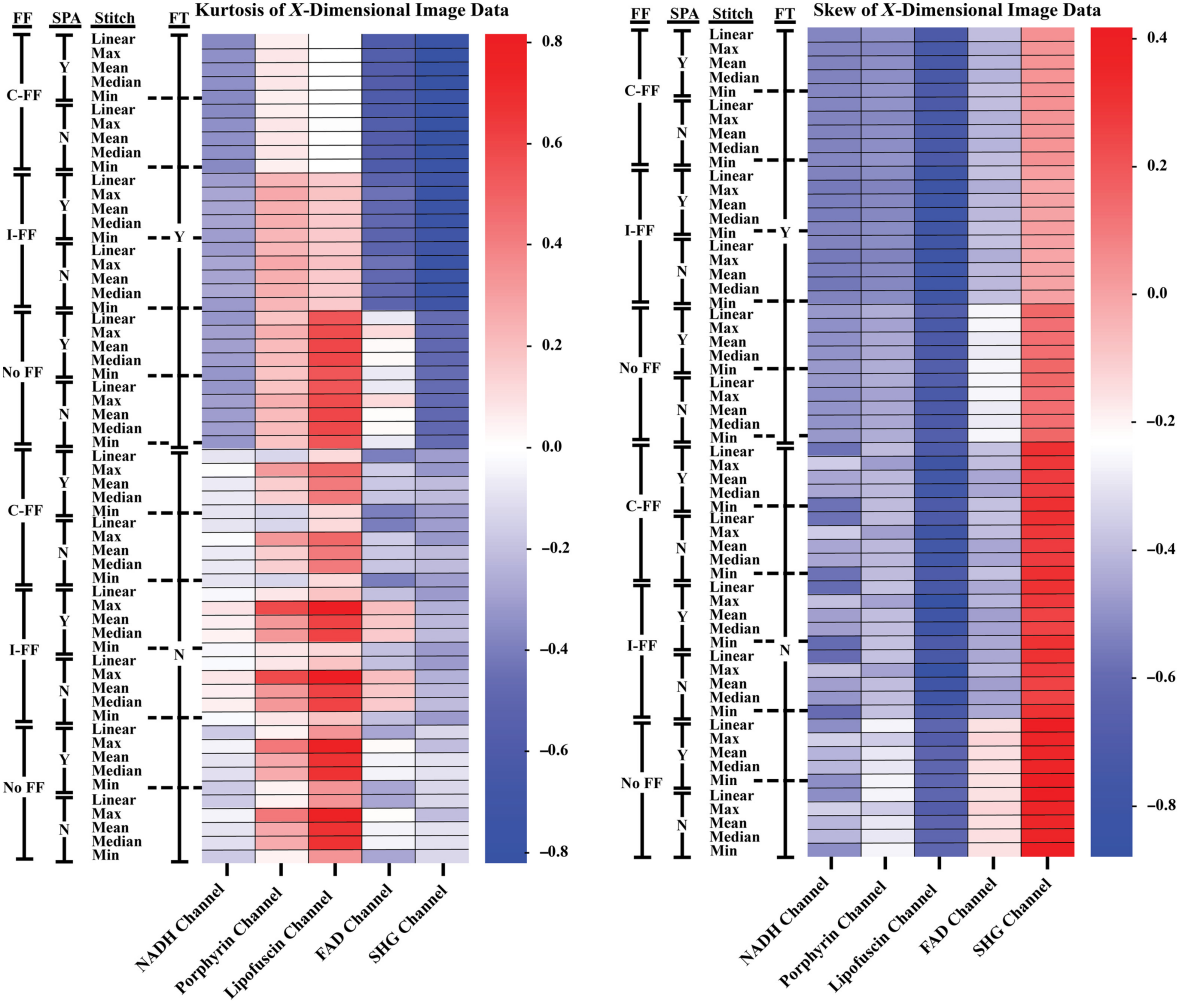
Heatmap representations of the kurtosis and skew of image data that has been averaged over the X-dimension (averaged over all rows in an image, ignoring values=0). SPA, linear interpolation used during the process of tile registration and stitching; C-FF, tiles corrected with the cumulative FF; I-FF, tiles corrected with the FF created from the individual z-stack images; No-FF, tiles not corrected with a FF; Y, FT filter process used; N, FT filter process not used.

**Fig. 6:**
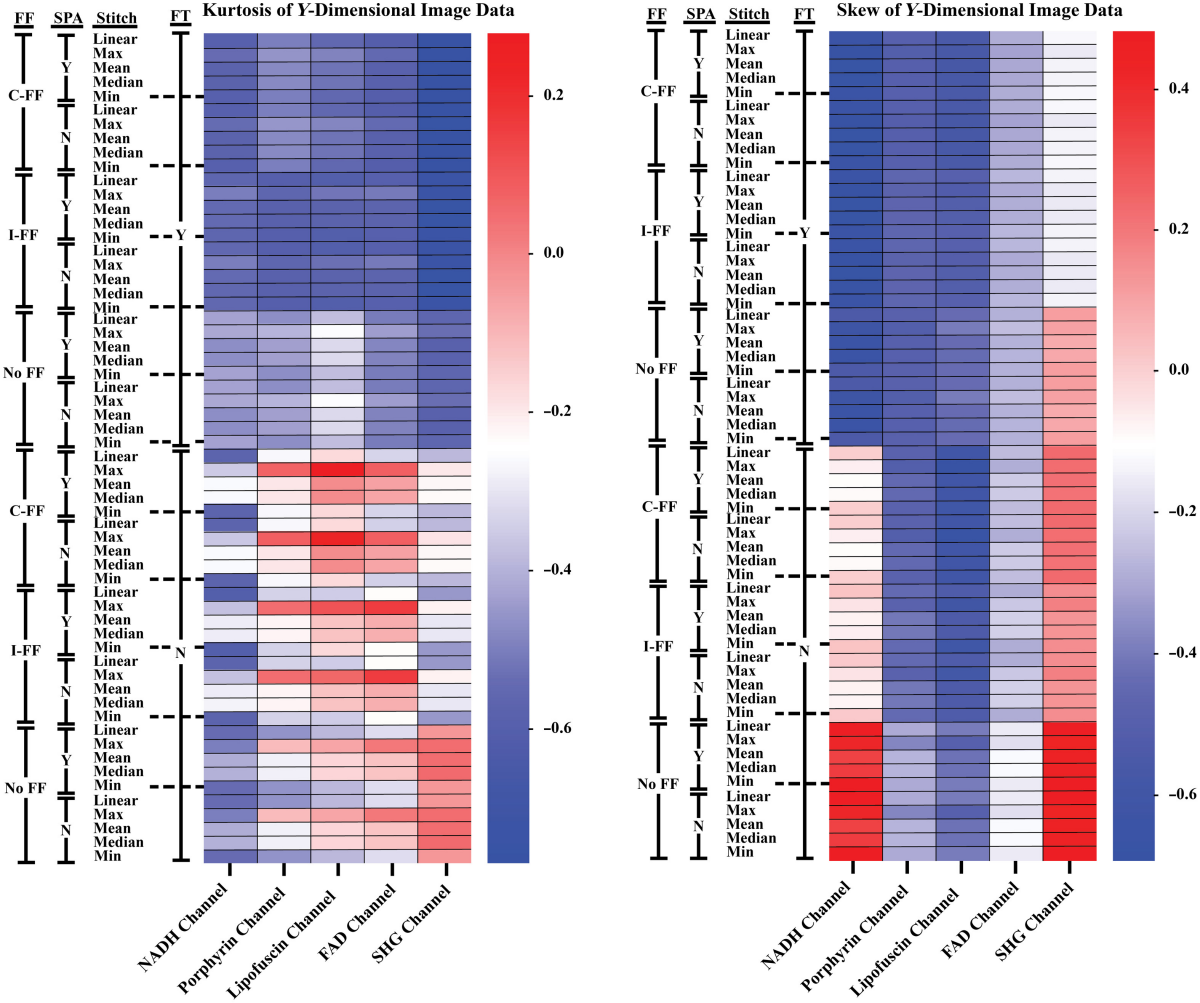
Heatmap representations of the kurtosis and skew of image data that has been averaged over the Y-dimension (averaged over all rows in an image, ignoring values=0). SPA, linear interpolation used during the process of tile registration and stitching; C-FF, tiles corrected with the cumulative FF; I-FF, tiles corrected with the FF created from the individual z-stack images; No-FF, tiles not corrected with a FF; Y, FT filter process used; N, FT filter process not used.

Because all image channels were collected using the same parameters besides excitation and detection wavelengths, they can be used to represent different degrees of image brightness and tile vignetting that stem from the autofluorescent properties of the tissue samples. In general, there was a trend of increasing brightness in the NADH, porphyrin, FAD, and the lipofuscin channels, respectively. The difference in channel intensities is likely a result of relative autofluorophore abundance and wavelength-dependent properties of the system hardware. Based on the trends seen in the kurtosis and skew of the image histograms averaged over the X-dimesion (Fig. 5), these processing methods are more capable of normalizing image brightness, i.e., reducing vignetting within the individual image tiles, for images that are within a range of vignetting severity. The comparison of a sample imaged with the NADH channel and lipofuscin channel (Fig. S3 in the Supplementary Material) exemplifies the range of vignetting that occurred in the sample images. The ability to improve normality of the image data was directionally dependent, as seen in the comparison between the kurtosis and skew of the X and Y dimensions. In general, increased processing broadened the distribution of pixel values in the Y-dimension (Fig. 6). With unequal vignetting, the statistics-based FF is biased toward the regions of reduced light transmittance. This bias appears to be slightly increased in the cumulative-FF approach as shown in Fig. 6 heatmap. Figure S3 in the Supplementary Material provides an example of uneven vignetting, which has influenced the variation seen between Figs. 5 and 6. The use of linear interpolation during the tile registration and stitching process does not appear to have a significant impact on quantitative or qualitative image quality retention or brightness correction.

**Fig. 7:**
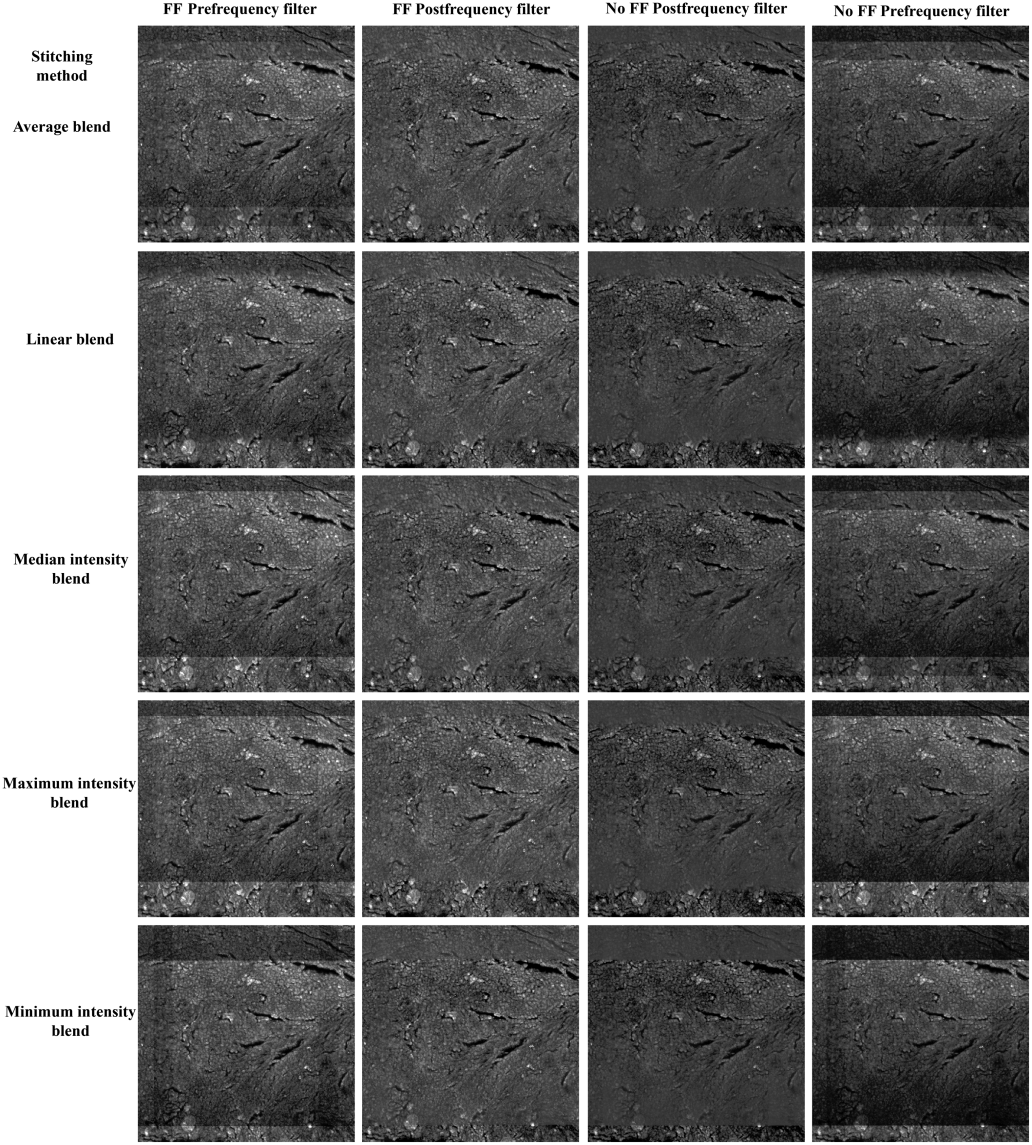
Qualitative comparison of changes in tiling artifacts using each stitching method, with and without FF correction, and pre-/postfrequency filter. There is a significant improvement in the tiling artifact between the pre-/postfrequency filtered images, with residual striping at the bottom of the images in the non-FF corrected images.

**Fig. 8:**
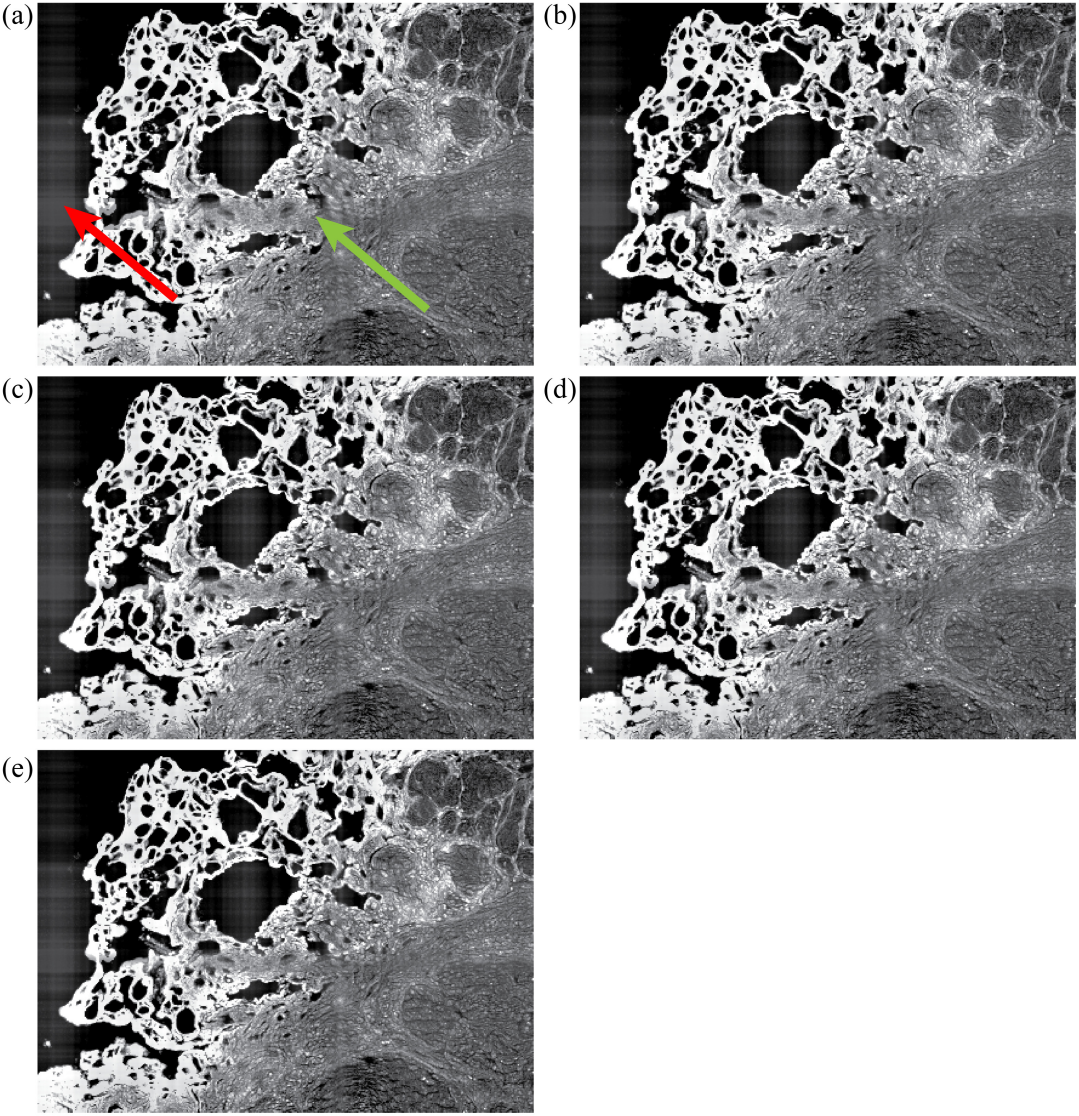
Further qualitative comparisons of cumulative-FF corrected images that have undergone FT filtering. (a) Tiles combined using the mean value, (b) linear combination, (c) maximum, (d) minimum, and (e) median. The red arrow in panel (a) indicates a “haloing” artifact that appears near tissue edges because of the FT filter. The green arrow in panel (a) indicates a line of blurring at the border of image tiles that occurs during the tile stitching process and varies between the different methods.

Qualitative comparisons in Fig. 7 show the FF-based correction does improve artifact smoothing by correcting for vignetting that occurs within the individual tile scan regions. The use of an FT filter helps mitigate the bias of the FF and smooth residual boundaries between adjacent tiles. Greater degrees of vignetting tend to result in more apparent boundaries between image tiles with the use of maximum, median, and minimum stitching methods. In certain instances, this can improve the correction of brightness nonuniformity using the FT filter as these boundaries represent greater image frequencies that are more easily removed by masking frequency values. While the FT filter helps to smooth residual boundaries between stitched tiles, it does introduce a haloing artifact that is most prominent around the high frequency boundaries of the tissue and background [Fig. 8(a)]. This is most prominent in the SHG images due to the high frequency image information that has an almost binary-like appearance. This could be reduced, or possibly completely removed, with a more careful approach toward adjusting the Fourier domain of the images. For example, instead of completely masking frequency values, a method of adjusting them to equalize nonuniformity in the image could prevent the removal of frequencies that produce smearing around object edge regions. The different methods of tile stitching introduce a varying degree of blur and/or deviation in brightness at the tile borders (Figs. 7 and 8). The slight improvement in the RMSE and SSIM quality metrics (Fig. 4) for the median and mean methods of combining overlapping regions of tiles is generally recapitulated in qualitative assessment of images. Effectiveness of processing is influenced by the degree of vignetting in the original images as shown by the variations seen between the separate image channels. The NADH channel, having the most severe brightness nonuniformity, is more normalized in the direction of vignetting (a gradient from top to bottom of the image tiles, Fig. 5), but is blurred by the FT filtering step [Fig. S3(c) in the Supplementary Material]. The difference in vignetting that occurs in the separate channels is due to a combination of tissue composition, fluorophore characteristics, and the optics of the imaging system. For example, lipofuscin is typically found in abundance within certain regions tissue, resulting in a greater signal emittance from these regions for the channel tuned to collect its fluorescence. Due to chromatic dependence of optical components in the microscope, images may have differences in overall brightness and field-dependent brightness variations, which may manifest as the correction schemes better or worse. These effects can be reduced at the time of acquisition by modifying parameters such as gain, frame averaging, dwell time, and magnification. To mitigate confounding variables, we kept our imaging parameters the same for each image channel, resulting in images that contained regions of over-/undersaturated pixels. This had the added advantage of allowing us to test the processing steps over a range of image qualities, although this study is still limited in that only a single tissue type was used.

## 4 Conclusion

Whole-slide, or large-scale, microscopy scans produce unique image datasets that aid in biomedical research applications. MPM is well known for its ability to image at a greater depth into samples, in comparison with confocal microscopy,^5^ and acquire label-free images such as the autofluorescence and SHG images used in this study. The combination of MPM with a tile or line scanning process has been used to study large biological samples^27^^–^^29^ and cultures^30^ that would otherwise require sacrificing information with smaller images or necessitate multiple image acquisitions. The ability to capture the entire sample within a single image provides the ability to quantify and compare points of interest in situations where it would otherwise be impossible. These same benefits of large-scale MPM imaging also apply to its use in the clinical setting as the technology continues to improve and is adopted into practice.^31^^,^^32^ Unfortunately, numerous alterations in hardware or tissue sample can result in uneven light capture during acquisition which results in line, or tiling, artifacts in the final composite image of these scans. To facilitate our microscopy research of human tissue, we have studied the effectiveness of various image processing techniques on eliminating these tiling artifacts and how they modify the original data.

Eleven samples of human duodenal tissue were imaged with an automated tile scanning method. The individual tiles from each sample were processed with statistics-based FF meshes, combined using various fusion methods, and filtered by masking frequency values in the Fourier domain. RMSE and SSIM were measured to determine retention of image data by comparing the images at differing processing stages to their raw image counterpart. We found that regions of the images not targeted by these processing steps, i.e., areas without the tiling artifact, are generally unmodified beyond improvements in the brightness nonuniformity between tile scans. This, however, does not hold for all image channels. Images with severe vignetting are only mildly improved or worsened through blurring. This could be alleviated by modifying the initial collection of data, e.g., by adjusting gain or increasing magnification to reduce vignetting. For image channels with a mild to moderate degree of vignetting, the cumulative FF correction appears to improve brightness uniformity in the direction of the vignetting when used in conjunction with FT filtering. This assumes that a more normal distribution of pixel values represents improved uniformity (Fig. 5). Because the FF is generated from the covariance of a line fit to the pixel values from each tile scan, a greater averaging of these values, as seen in the cumulative FF approach, is believed to provide a better representation of the abnormal transmission of light occurring during the imaging process.

Modifications to these methods are necessary for general applications. First, further study of how these methods work for MPM images of different tissue types would provide a better representation of its general use. The variations in performance seen between the different image channels suggests that an approach that is modified based on the input image would function better for multiwavelength datasets. It is possible that the trends found in this study would change based on tissue characteristics, e.g., through differences in frequency information of the tissue itself that could be affected by the FT filter, posing a potentially significant limitation to this study. Two primary targets for improvement of the artifact removal process are how the FF mesh is generated, e.g., finding and fitting different functions to the pixel values, and how values in the Fourier domain are changed. The inclusion of machine learning could help to realize this optimization process and push it towards automation. Currently, the user can run a Python script to process and combine the image tiles and filter the frequency values of the full image. The use of machine learning could provide better oversight of this process by monitoring the changes that occur at each step. For example, a model could be trained on in-focus regions of the image that should occur within the central portions of each tile scan. This model could then assess the tile boundaries after they are combined to determine if the flat-fielding and/or stitching was appropriate. Another potential application of machine learning would be the rapid change and assessment of the frequency information, i.e., modifications of the Fourier domain paired with analysis of the image artifacts to find an ideal transformation to eliminate the artifact appearance.

Another focus for future work is the analysis of how well biological information is retained postprocessing, specifically in the image regions that contain the tile artifacts. One method for comparing processed data to a ground-truth image would be to capture small 2×2 tile scans within a region that could be imaged within a single field of view. While this would allow for an almost one-to-one comparison, it would significantly increase the length of data acquisition and risk missing regions that would otherwise be captured in a large-scale tile scan as used in this study. Machine learning could be another potential solution for this analysis, for instance, with the use of a model that can discriminate between original and processed image data of interest.

Overall, the use of these methods provides a means of enhancing the quality of large-scale microscopy images in an easily accessible manner. Without the use of any “black box” processes or proprietary software, users can identify how the original data were modified using these steps and begin to incorporate it into their own microscopy research.

## Data Availability

The data presented in this article are not yet made publicly available. They can be requested from the author at tsawyer9226@arizona.edu.
