# Supplementary material for: Combined flat-field and frequency filter approach to correcting artifacts of multichannel two-photon microscopy
Source: J Biomed Opt. 2024 Jan 23;29(1):016007. doi: 10.1117/1.JBO.29.1.016007 (PMC10804314; doi:10.1117/1.JBO.29.1.016007)
Supplement: Supplementary file 1 [file JBO_029_016007_SD001.pdf]

Supplemental Material

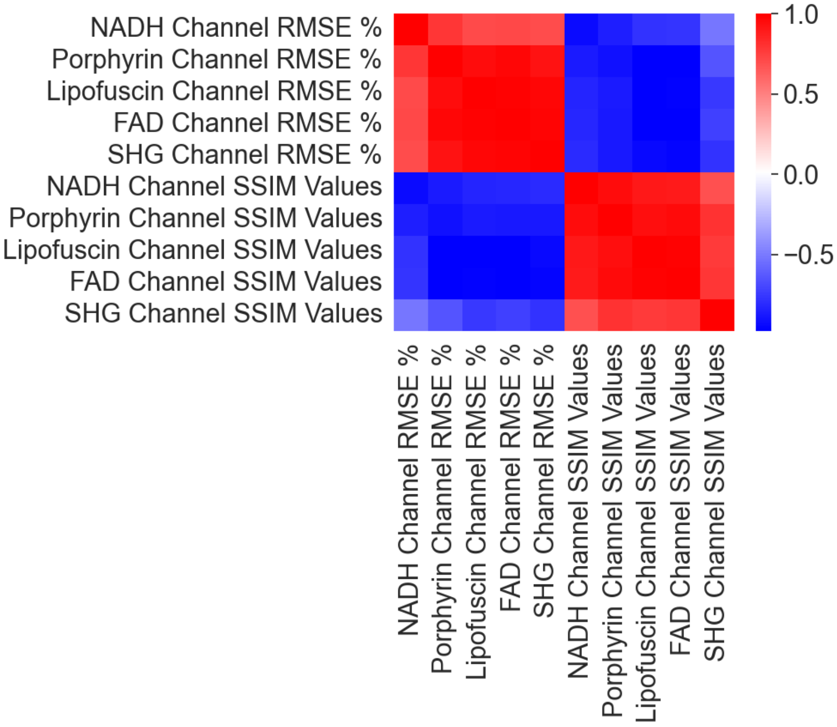

Fig S1 Correlation matrix of the RMSE and SSIM values derived from comparing the processed images to their raw counterpart.

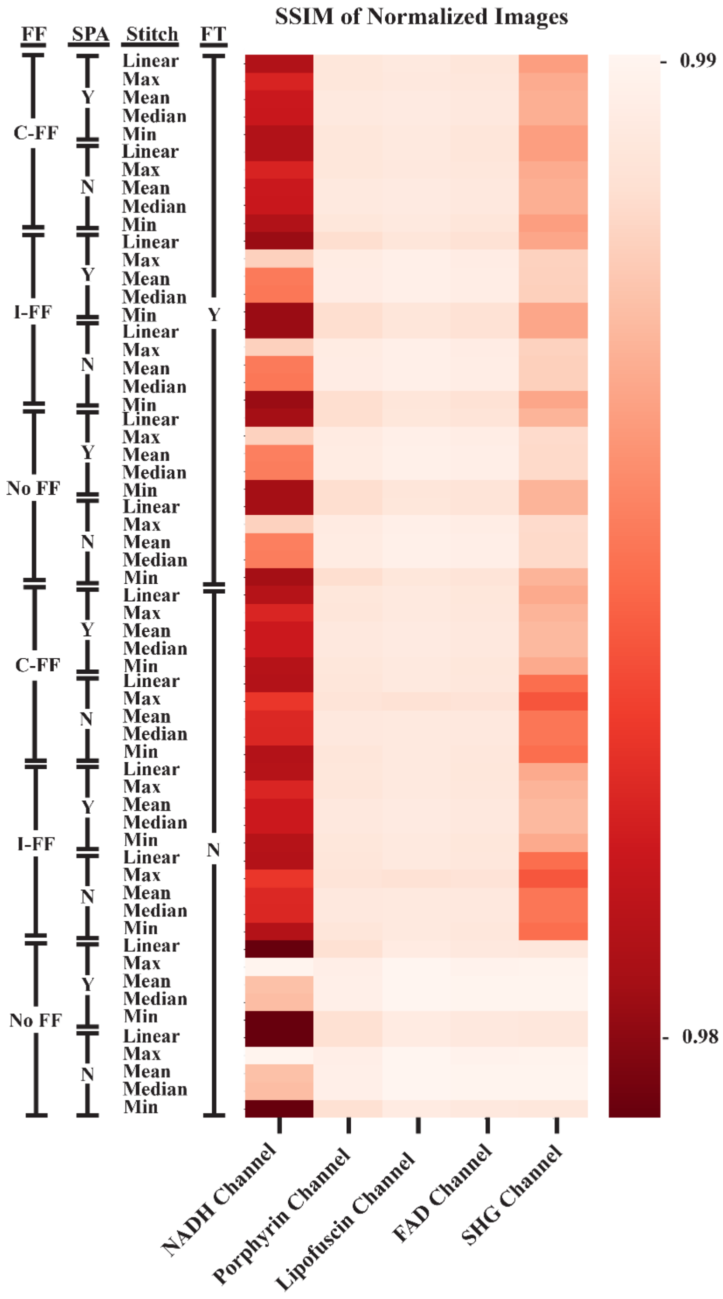

Fig. S2 The SSIM values of processed compared to raw images after image values have been normalized to floating point values between 0 – 1. Note the scalebar showing almost complete similarity pre/post-processing.

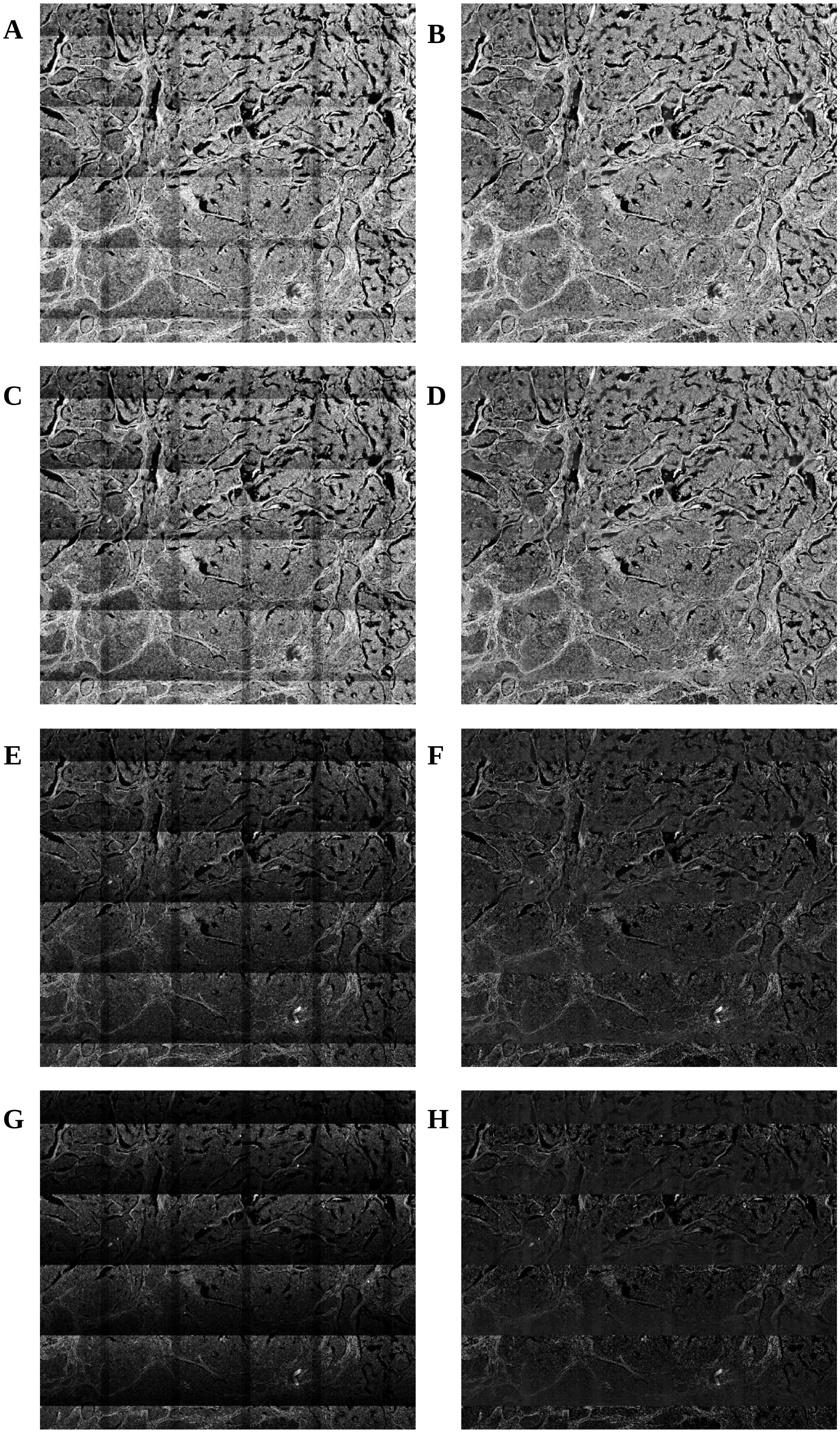
A
B
C
D
E
F
G
H

Fig. S3 Comparison of lipofuscin and NADH channels, which were generally the brightest and dimmest channels (respectively) at the set imaging parameters. (A) Lipofuscin image tiles that have been FF corrected and fused. (B) Post-FT filtering of (A). (C) Lipofuscin image tiles without FF correction. (D) Post-FT filtering of (C). (E) NADH image tiles that have been FF corrected and fused. (F) Post-FT filtering of (E). (G) NADH image tiles without FF correction. (H) Post-FT filtering of (G).

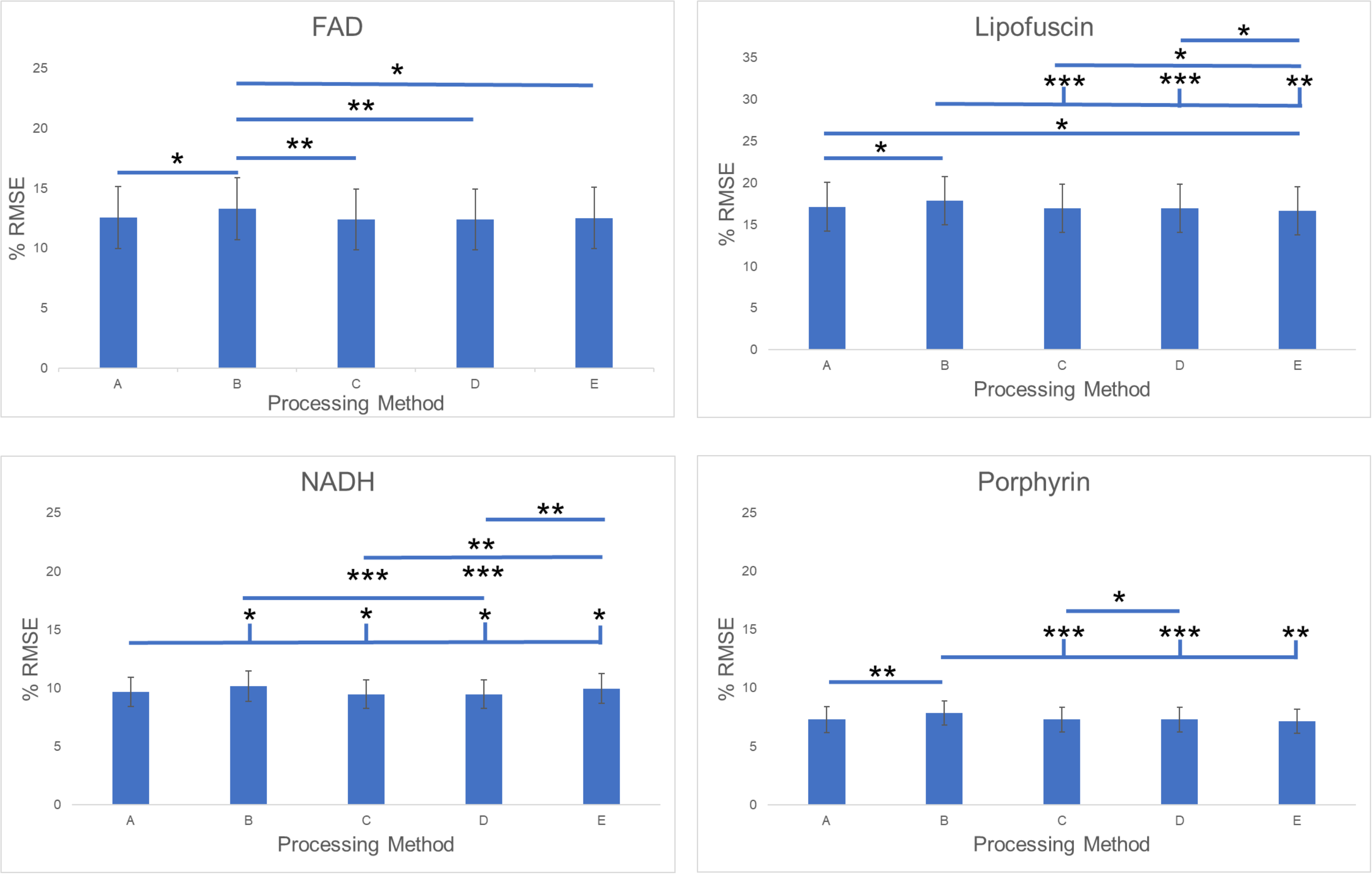

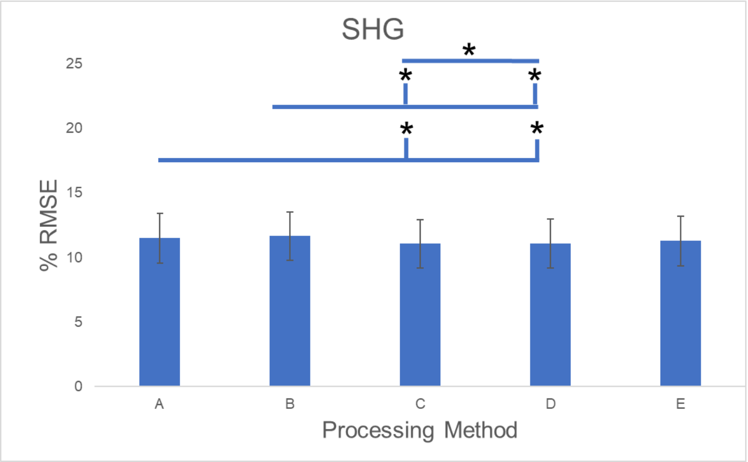

Fig. S4 Comparisons of the percent RMSE at the highest level of processing for each imaging channel. *Each image in this comparison has been processed with cumulative flat-fielding and frequency filtering, differing in the tile combination method as follows:* A – linear blending of overlapping pixel values, B – Maximum pixel values, C – Mean of the pixel values, D – Median of the pixel values, E – Minimum pixel values. Each method was compared using a two-tailed, paired, t test. * = $p < 0.05$, ** = $p < 0.001$. *** = $p < 0.0001$. Error bars show the calculated standard error of the measurement from the population of eleven image samples.

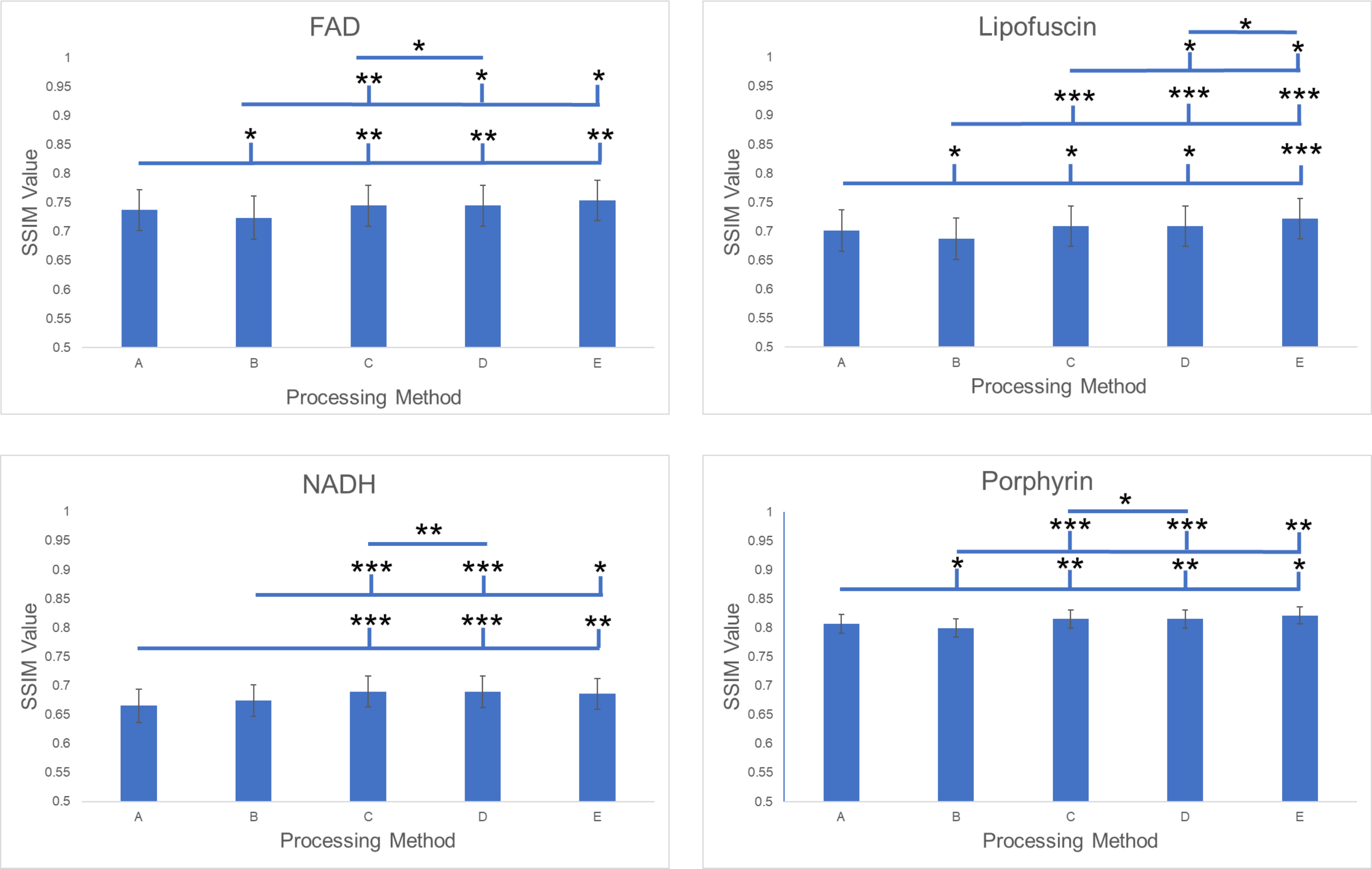

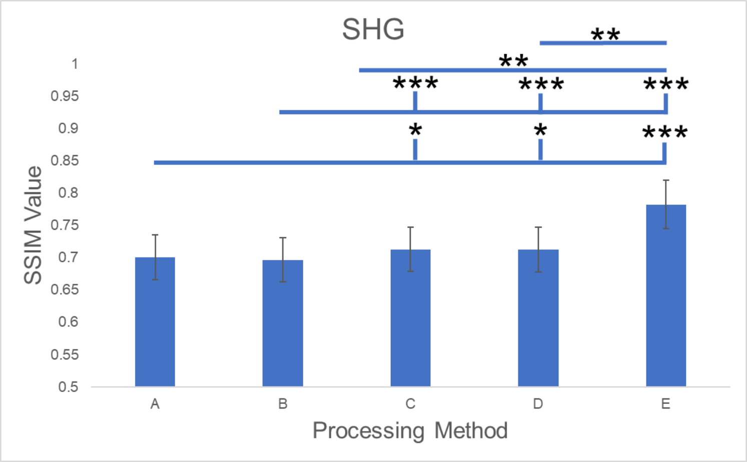

Fig. S5 Comparisons of the SSIM values at the highest level of processing for each imaging channel. *Each image in this comparison has been processed with cumulative flat-fielding and frequency filtering, differing in the tile combination method as follows:* A – linear blending of overlapping pixel values, B – Maximum pixel values, C – Mean of the pixel values, D – Median of the pixel values, E – Minimum pixel values. Each method was compared using a two-tailed, paired, t test. * = $p < 0.05$, ** = $p < 0.001$. *** = $p < 0.0001$. Error bars show the calculated standard error of the measurement from the population of eleven image samples.

FAD RMSE				
Linear stitch C-FF FT-filtered	Max stitch C-FF FT-filtered	Mean stitch C-FF FT-filtered	Med stitch C-FF FT-filtered	Min stitch C-FF FT-filtered
13.60745883	14.20710836	13.09344466	13.10713426	13.62471275
5.149976392	6.4241032	5.365484355	5.362320876	4.850445156
10.09509076	11.70310381	10.37570644	10.3778905	10.05890571
8.126312834	9.158246486	8.163368001	8.161180836	7.493718407
5.263518915	5.777546663	5.233688134	5.234535925	5.433246723
5.840368789	6.391657776	5.930693418	5.928654839	5.719726377
12.44553882	14.38126797	12.91482019	12.91650966	12.8274035
35.4045156	36.91425575	35.14363126	35.16535019	34.99550195
17.04405887	16.13857021	15.81873855	15.84828671	17.36658355
14.63309316	13.79108828	13.93582004	13.94151404	14.36554518
10.67232585	11.52322541	10.6090834	10.60938904	10.99114164

Lipofuscin RMSE				
Linear stitch C-FF FT-filtered	Max stitch C-FF FT-filtered	Mean stitch C-FF FT-filtered	Med stitch C-FF FT-filtered	Min stitch C-FF FT-filtered
16.46183472	17.20263004	15.89972843	15.9433386	15.55967443
7.981651783	8.377038668	7.626894026	7.631298759	7.524807073
11.1482036	13.19399453	11.64338297	11.64431894	10.40392973
24.67596038	25.6276066	24.42857009	24.43616143	24.20192611
6.624628258	7.564299959	6.815618603	6.815610647	6.832354839
23.23258473	23.63081479	23.14176641	23.1465019	22.95630988
7.973308261	9.225149045	8.370625922	8.364767499	7.913364507
37.96203181	38.84869954	37.62905032	37.64569299	37.52354481
17.17143517	17.40023361	16.44443738	16.49773267	15.82528442
25.01745839	23.94727687	24.14459407	24.14902149	23.68411963
10.37763034	11.50667309	10.38592837	10.3866116	10.66581323

NADH RMSE				
Linear stitch C-FF FT-filtered	Max stitch C-FF FT-filtered	Mean stitch C-FF FT-filtered	Med stitch C-FF FT-filtered	Min stitch C-FF FT-filtered
9.1579356	9.605174306	8.909653197	8.919743202	9.464101659
12.62946596	13.51790827	12.49483971	12.49551741	13.36100083
11.32135498	12.42182716	11.46281077	11.45872434	11.40478255
9.515553562	10.02029842	9.307295681	9.306442795	9.621274333
3.485937437	3.770280417	3.442462194	3.442660074	3.664827123
16.37755115	17.58382389	16.19859208	16.20625865	16.50356767
5.691354501	5.681660425	5.491589453	5.494432934	5.722744388
9.581976464	9.510090832	9.063402493	9.068031996	10.05915247
14.88184897	15.08783994	14.42850377	14.45473191	15.46513039
4.051024691	4.174041222	3.884325037	3.886641011	4.26819373
9.582508237	10.48485709	9.57142376	9.575733126	10.08159939

Porphyrin RMSE				
Linear stitch C-FF FT-filtered	Max stitch C-FF FT-filtered	Mean stitch C-FF FT-filtered	Med stitch C-FF FT-filtered	Min stitch C-FF FT-filtered
6.54406545	7.667352912	6.739855307	6.743677231	6.785703045
3.961065987	4.537503361	3.917781342	3.917582086	3.852430192
4.271277259	4.924132723	4.417296228	4.418561197	4.45849152
10.83785074	11.09491368	10.62087919	10.62076354	10.16975474
2.688630722	3.051274928	2.733853964	2.733571588	2.851661067
9.688826854	10.0445803	9.622879749	9.624665075	9.335139853
4.992895258	5.867878506	5.204800236	5.204687733	5.222723453
14.53391785	14.64709123	14.37739796	14.38066774	14.44611998
6.765162567	7.648195589	6.839179682	6.842663305	6.62133013
10.51975209	10.68271688	10.28238345	10.28475797	9.130196559
5.476393906	6.168310501	5.505211069	5.505761316	5.743620934

SHG RMSE				
Linear stitch C-FF FT-filtered	Max stitch C-FF FT-filtered	Mean stitch C-FF FT-filtered	Med stitch C-FF FT-filtered	Min stitch C-FF FT-filtered
20.0816922	20.91424127	20.02172718	20.06655153	20.82862514
5.547366302	6.954347166	5.61141646	5.607719875	5.320048673
13.26669815	12.39066838	12.33943259	12.34619203	12.78325669
5.8759805	6.397443055	5.518684857	5.520051214	5.648721883
7.449414224	7.479438598	7.163344459	7.165532307	7.249587918
8.492455227	9.194602098	8.178985015	8.195717241	8.360273007
6.734474604	7.045224895	6.574485988	6.576201359	6.421632882
21.9496105	22.69889152	21.52778677	21.54923019	21.55086711
15.25787736	14.33403382	14.1923297	14.20198806	14.95693136
4.238138732	4.129666226	3.978741552	3.983968127	4.213687041
17.55908251	16.59778295	16.57934188	16.5846866	16.74010937

FAD SSIM				
linear stitch Agg-FF FT-filt	max stitch AggFF FTfilt	mean stitch AggFF Ftfilt	med stitch AggFF Ftfilt	min stitch AggFF FTfilt
0.822406247	0.810246422	0.828875549	0.828754112	0.840428325
0.78950053	0.755959415	0.788852858	0.788831528	0.818308287
0.775565123	0.759995183	0.78588911	0.785803684	0.785540978
0.833121209	0.815550689	0.834775847	0.834734043	0.861828464
0.805875775	0.798107487	0.814320878	0.814195801	0.822497923
0.839067472	0.832995244	0.847293958	0.847371277	0.860667298
0.719649725	0.698286822	0.723164227	0.723119421	0.727082028
0.435315863	0.390263278	0.438328238	0.438081689	0.467669383
0.642106135	0.648711728	0.658952312	0.658615043	0.64617848
0.732371011	0.7424181	0.746763324	0.746476852	0.744446011
0.716480501	0.709782535	0.728155863	0.728052576	0.717091986

Lipofuscin SSIM				
Linear stitch C-FF FT-filtered	Max stitch C-FF FT-filtered	Mean stitch C-FF FT-filtered	Med stitch C-FF FT-filtered	Min stitch C-FF FT-filtered
0.765320275	0.753430602	0.772805463	0.772124399	0.793715833
0.806951727	0.789059039	0.81317528	0.812872814	0.829017013
0.801903015	0.762098993	0.799214441	0.799176377	0.818240862
0.70455727	0.675554614	0.702096996	0.70207346	0.736656567
0.779341255	0.770366386	0.788761576	0.788656821	0.802387385
0.742381336	0.725569596	0.746198533	0.745999464	0.752800761
0.69142548	0.694203502	0.709482788	0.709421256	0.706513282
0.382670203	0.361162123	0.396349124	0.395804805	0.413335232
0.644489648	0.63948454	0.656084423	0.655841284	0.665604761
0.664340514	0.65758906	0.668087771	0.668004194	0.686941122
0.731561308	0.72542171	0.743638438	0.743525994	0.735488819

NADH SSIM				
Linear stitch C-FF FT-filtered	Max stitch C-FF FT-filtered	Mean stitch C-FF FT-filtered	Med stitch C-FF FT-filtered	Min stitch C-FF FT-filtered
0.741225685	0.755390738	0.765599995	0.764833731	0.754165183
0.696047369	0.708030195	0.723772129	0.723709578	0.713005628
0.612791317	0.636089113	0.64857257	0.648184893	0.628244296
0.796692013	0.816448504	0.821047882	0.820764271	0.81479349
0.723105734	0.705015324	0.732682038	0.732639853	0.748286567
0.717108432	0.71043603	0.733846658	0.733582295	0.718577984
0.55412386	0.561443436	0.575922087	0.57558742	0.588301907
0.572875196	0.594936847	0.610874285	0.610299	0.62123346
0.559316815	0.581026005	0.592088209	0.591633589	0.584990413
0.7827336	0.77467409	0.79317017	0.793074604	0.794666865
0.560915907	0.571704258	0.589766844	0.589183524	0.574857937

Porphyrin SSIM				
Linear stitch C-FF FT-filtered	Max stitch C-FF FT-filtered	Mean stitch C-FF FT-filtered	Med stitch C-FF FT-filtered	Min stitch C-FF FT-filtered
0.871047236	0.865425136	0.877661817	0.87745614	0.873980963
0.843475694	0.823789443	0.847608838	0.847500428	0.860475653
0.821538675	0.812903643	0.828665443	0.828498917	0.820731755
0.856575488	0.853778748	0.865345004	0.865246332	0.881543568
0.84013347	0.827485454	0.844905385	0.844839859	0.84746039
0.840414515	0.833746872	0.848642708	0.848518548	0.850484806
0.784490818	0.770589754	0.788223856	0.78820751	0.788463534
0.678955103	0.686282902	0.700226413	0.699713599	0.724410219
0.770467674	0.761306278	0.779261132	0.778979593	0.788014867
0.796699347	0.795569467	0.803924486	0.803860173	0.828991229
0.769824855	0.765614533	0.781319663	0.781263216	0.767285262

SHG SSIM				
Linear stitch C-FF FT-filtered	Max stitch C-FF FT-filtered	Mean stitch C-FF FT-filtered	Med stitch C-FF FT-filtered	Min stitch C-FF FT-filtered
0.498184528	0.494337271	0.497182022	0.497001462	0.487804022
0.753036355	0.727259083	0.761165399	0.760971389	0.857631421
0.660082988	0.660505416	0.678782401	0.678715813	0.796877944
0.842662875	0.841391368	0.854542302	0.854366788	0.905117749
0.739366939	0.737221803	0.751864333	0.751602981	0.862943661
0.815404629	0.816078521	0.830050062	0.829802972	0.875669807
0.715540598	0.689533483	0.714914267	0.714933625	0.811917179
0.607137729	0.594386017	0.616123255	0.616567727	0.691011735
0.622774413	0.63981221	0.65187652	0.651463011	0.732101184
0.862311224	0.856473235	0.868958676	0.868755199	0.894668397
0.593088548	0.602135768	0.617400293	0.617122491	0.688972916

Tables S6 – S15 Raw data of the quality metrics measured from samples that underwent the highest degree of processing to remove tiling artifacts (the same data used to generate the plots in S4 and S5). From the earlier plots and this raw data, we observe obvious variations in the metrics between channels and separate samples.

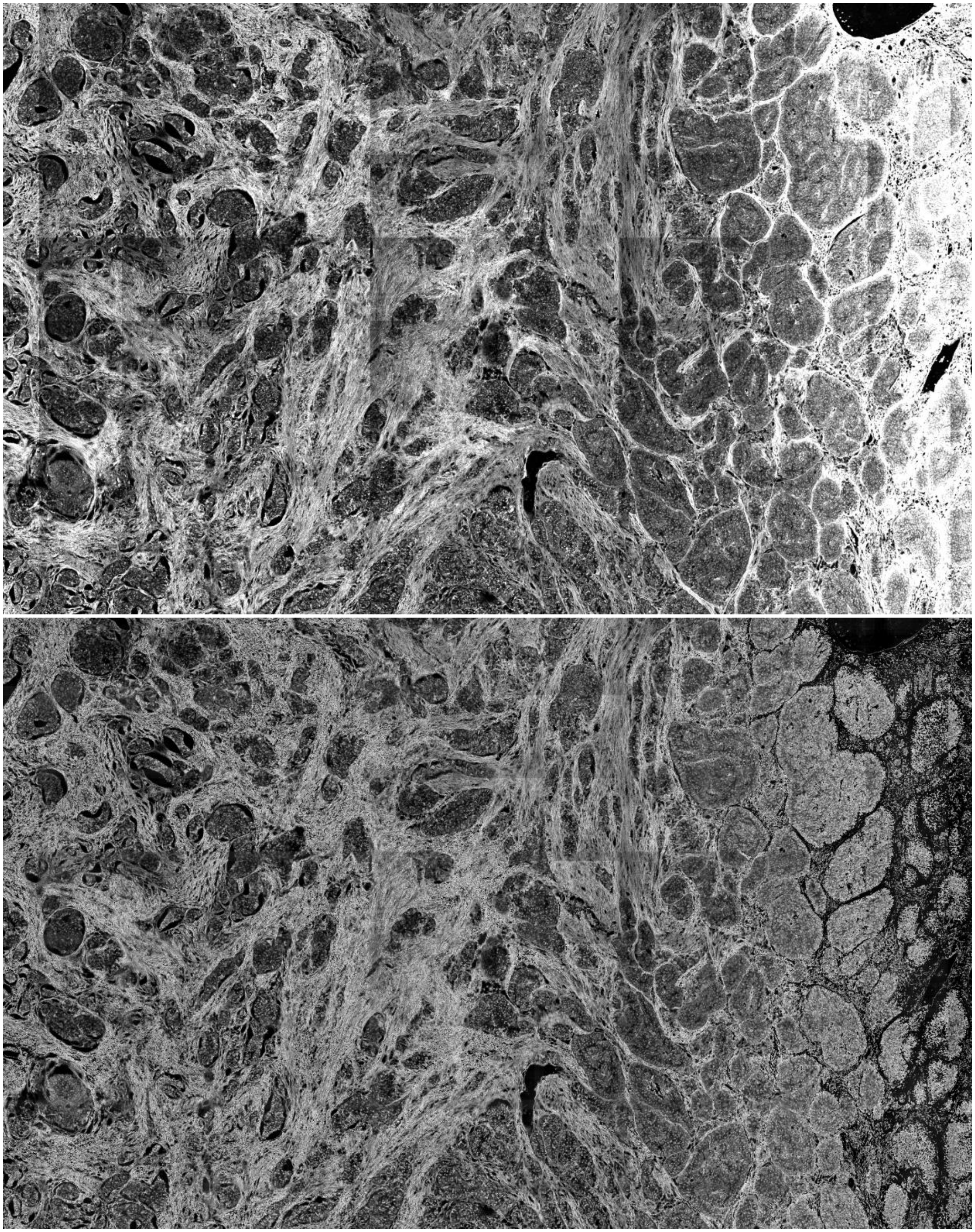

Fig. S16 Comparison of pre (top) and post-processed (bottom) images from the sample that resulted in the worst quality metrics for the majority of imaging channels in both the % RMSE and SSIM measurements. Regions of over-saturation seen on the right side of the original image likely had the largest contribution to this observation of the quality metrics.

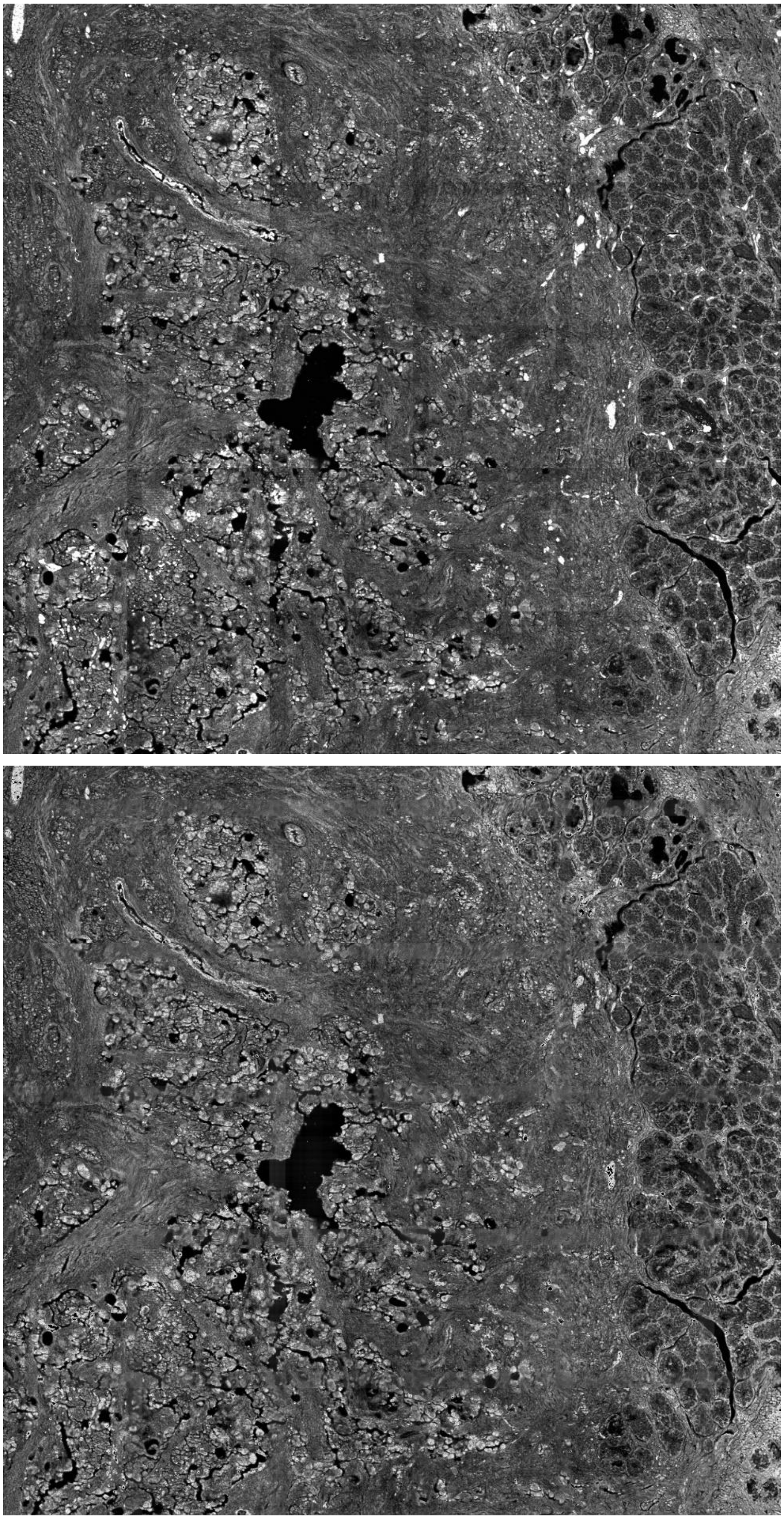

Fig. S17 Comparison of pre (top) and post-processed (bottom) images from a sample that had, in general, some of the best % RMSE and SSIM values. While the processing methods can help correct wide variations of brightness non-uniformity (seen in the transformed regions of over-saturation in **S16**), the RMSE and SSIM quality metrics are deficient in quantifying 'correctness' of the final image. For this example, the more uniform brightness of the original image resulted in less of a deviation of pixel values during processing which contributed to the perceived increase in performance based on the quality metrics.
